# Role of Long-Range Protein Dynamics in Different Thymidylate Synthase Catalyzed Reactions

**DOI:** 10.3390/ijms16047304

**Published:** 2015-04-01

**Authors:** Thelma Abeysinghe, Amnon Kohen

**Affiliations:** Department of Chemistry, University of Iowa, Iowa City, IA 52242-1727, USA; E-Mail: donthelma-abeysinghe@uiowa.edu

**Keywords:** TSase, KIEs, kinetic isotope effects, WT, wild-type, TRS, tunneling ready state

## Abstract

Recent studies of *Escherichia coli* thymidylate synthase (*ec*TSase) showed that a highly conserved residue, Y209, that is located 8 Å away from the reaction site, plays a key role in the protein’s dynamics. Those crystallographic studies indicated that Y209W mutant is a structurally identical but dynamically altered relative to the wild type (WT) enzyme, and that its turnover catalytic rate governed by a slow hydride-transfer has been affected. The most challenging test of an examination of a fast chemical conversion that precedes the rate-limiting step has been achieved here. The physical nature of both fast and slow C-H bond activations have been compared between the WT and mutant by means of observed and intrinsic kinetic isotope effects (KIEs) and their temperature dependence. The findings indicate that the proton abstraction step has not been altered as much as the hydride transfer step. Additionally, the comparison indicated that other kinetic steps in the TSase catalyzed reaction were substantially affected, including the order of the substrate binding. Enigmatically, although Y209 is H-bonded to 3'-OH of 2'-deoxyuridine-5'-mono­phosphate (dUMP), its altered dynamics is more pronounced on the binding of the remote cofactor, (6*R*)-*N*^5^,*N*^10^-methylene-5,6,7,8-tetrahydrofolate (CH_2_H_4_folate), revealing the importance of long-range dynamics of the enzymatic complex and its catalytic function.

## 1. Introduction

The role of protein dynamics in enzyme catalysis is one of the open questions in enzymology today. In particular, the participation of residues distal to the active site in catalyzing bond-activation at the active site is a topic of interest as it combines several enigmas: the long range communication in the enzyme, its “holistic” nature, and the different catalytic strategies applied by the same active site to catalyze different chemical conversions. Recent experimental evidence suggests that mutations distant from the active site affect chemical bond activations in numerous enzymatic studies including dihydrofolate reductase [1,2,3,4], human purine nucleoside phosphorylase [5,6], and soybean lipoxygenase-1 [7].

Thymidylate synthase (TSase, EC 2.1.1.45) catalyzes the synthesis of 2'-deoxythymidine-5'-mono­phosphate (dTMP), using (6*R*)-*N*^5^,*N*^10^-methylene-5,6,7,8-tetrahydrofolate (CH_2_H_4_folate) as a cofactor and 2'-deoxyuridine-5'-mono-phosphate (dUMP) as the substrate [8]. The essential role of this enzyme in *de novo* synthesis of a precursor of DNA, thymidine, has made this enzyme an outstanding target for the development of antiproliferative therapeutics for several decades [9,10]. In fact, one of the most commonly used pyrimidine analog drugs in the treatment of cancer is 5-fluorouracil, which is a covalent inhibitor of TSase [10]. Due to its biological and pharmacological importance, TSase has been studied kinetically and structurally over many years in many theoretical and experimental studies [8,11,12,13].

**Figure 1:**
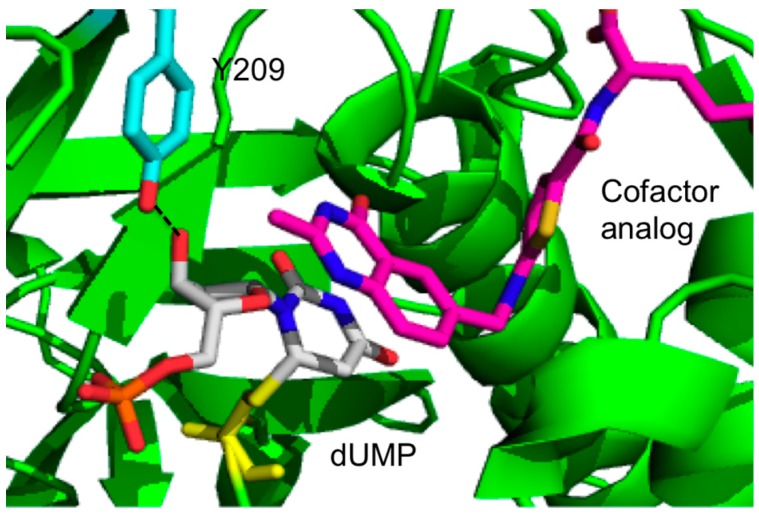
Active site crystal structure of the wild type (WT) *ec*TSase (green; PDB ID 2KCE) with 2'-deoxyuridine-5'-mono-phosphate (dUMP) (gray) and CH_2_H_4_folate analog, Raltitrexed (magenta). Tyr209 is shown in cyan. H-bond between 3'-OH of the dUMP and Tyr209 is shown in dashed lines. TSase chain is shown in cartoon representation (green). Residue Y209, the ligands dUMP and the cofactor analog, raltitrexed and the thio-ether bond between C-6 of dUMP and the C146 are in sticks. Color code for Y209: cyan (carbons), red (oxygen); Color code for dUMP: blue (nitrogen), grey (carbon), red (oxygen), orange (phosphorous); Color code for raltitrexed: blue (nitrogen), magenta (carbon), yellow (sulfur), red (oxygen), and the atoms in the thio-ether bond are shown in yellow.

Newby *et al.* have used kinetic and X-ray crystallography experiments to study the role of a highly conserved residue Y209 of *Escherichia coli* TSase (*ec*TSase), 8 Å away from where the chemistry takes place, which contributes one of only two hydrogen bonds to the ribosyl 3'-hydroxyl group of dUMP (Figure 1) [14].

**Figure 2:**
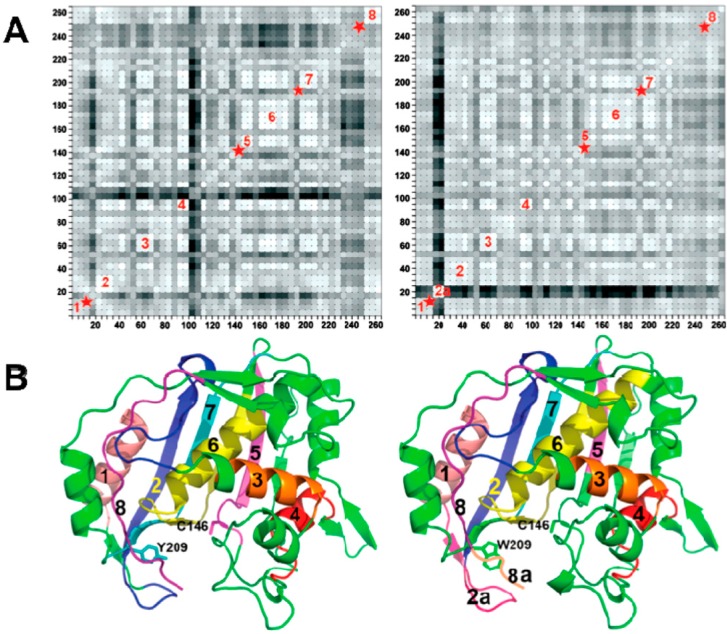
(**A**) The residue-based matrix plots showing correlations of anisotropic B-factor displacements for *Escherichia coli* TSase(*ec*TSase) atom pairs identified by the (*x*,*y*) grid coordinates of the plot. The left plot is for the WT *ec*TSase and the right plot is for Y209W *ec*TSase (Reproduced from [14] with permission from the American Chemical Society (ACS)). The degree of correlation is represented by color-coding, where the lighter shades of grey indicate greater correlation. The blocks of light-colored squares along the diagonals of the plots indicate the protein residues that vibrate as rigid bodies. The red stars indicate segments with disrupted rigid body vibrations of the Y209W mutation. Segment 2A in the right plot is the phosphate-binding loop with relatively higher B factors; (**B**) Ribbon diagrams of WT (**left**) and Y209W (**right**). The segments labeled in (**A**) are colored. The cofactor analog (CB3717), dUMP and the mutated residue and catalytic cysteine are shown as sticks. Segment 8A represents the *C*-terminal. (adapted from [15] with permission from the ACS).

Succinctly, these investigators used the crystal structures of the wild type (WT) and Y209W ternary complexes with dUMP and CB3717, an analogue of the cofactor (PDB IDs 2G80 and 2G8M, respectively). Those studies showed that the crystal structure of Y209W complex is strikingly similar to WT-dUMP-CB3717 at a resolution of 1.3 Å [14,15]. The most conspicuous difference between these two crystal structures was in the anisotropic B-factors, the refined anisotropic B factors of the crystal structures revealed that some protein segments of Y209W have disrupted rigid-body vibrations compared to the WT (Figure 2). Anisotropic B-factors provide the information of the directionality of the atomic mean square displacements in a given crystal. The anisotropic B factors of atoms in several loops across the WT protein are all oriented in the same direction. This suggests a concerted movement of these loops in the WT TSase. However, in Y209W, the anisotropic B factors of those segments (marked by red stars in Figure 2A) are randomly oriented, indicating a disruption of the correlated atomic vibrations of protein residues in the mutant.

The higher *K*_M_ of CH_2_H_4_folate is in accordance with the conformation of Y209W mutant prior to hydride transfer being more disordered and further away from the dUMP than in the WT. This is in agreement with the significant changes in the average B factors of several loops in Y209W crystal structure compared to the WT. Greater mobility of these loops could impair the binding of the substrate and cofactor. Subsequently, the effect of Y209W mutation on the mobility of this loop (segment 2a, the phosphate-binding loop) can propagate to the other regions in the active site cavity.

**Scheme 1:**
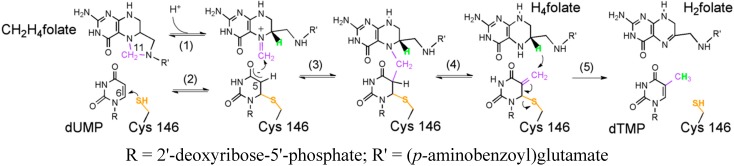
The proposed mechanism for thymidylate synthase (adapted from [17] with copyright permission from the ACS).

The substitution of tyrosine to tryptophan dramatically affected the catalytic rate and the *K*_M_ values for the substrate and cofactor. Interestingly, the trend was more pronounced for the cofactor of the enzyme, CH_2_H_4_folate, although its binding site is even more remote from the mutation’s site. The lower affinity for CH_2_H_4_folate indicates a need of modulating contacts between different segments of the protein. Furthermore, those findings suggest that Y209W mutation change protein dynamics that play a direct role in certain catalytic steps of TSase. However, the analysis performed in [14] did not identify the specific steps affected by the mutation. The mechanism of TSase (Scheme 1) involves several chemical conversions including two C–H bond activations: a rate limiting hydride transfer (step 5) and a much faster proton transfer (step 4). Both these H-transfers have been studied in the WT enzyme via examination of the temperature dependence of intrinsic kinetic isotope effects (KIE_intS_) [16].

**Figure 3:**
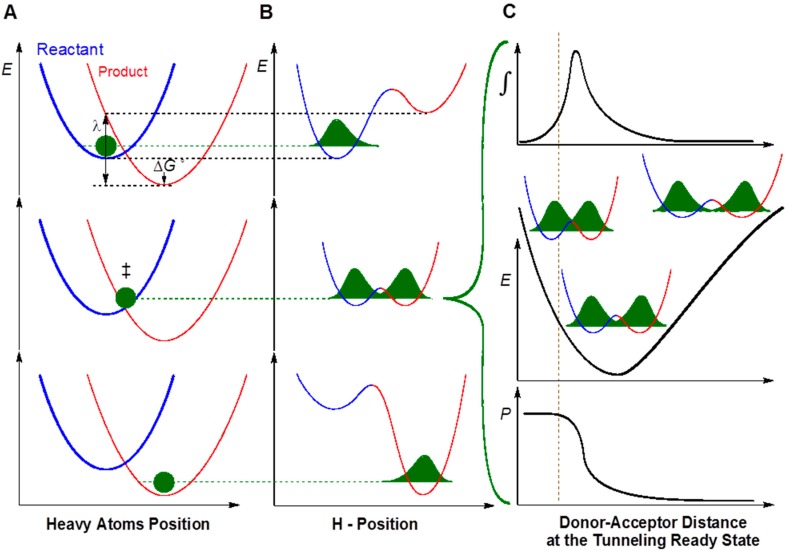
An example of a model used to analyze kinetic isotope effects (KIE) for H-transfer reactions (sometimes called Marcus-like models). Three slices of the potential energy surface (PES) along components of the collective reaction coordinate showing the effect of heavy-atom motions on the zero point energy in the reactant (blue) and product (red) potential well. (**A**) Presents the heavy atom coordinate (also known as the Marcus parabolas); and (**B**) shows the H-atom position, which is orthogonal to the heavy-atom coordinate. In the top panels, the hydrogen is localized in the reactant well, and the zero point energy of the product state is higher than that of the reactant state. Heavy atom reorganization brings the system to the tunneling ready state (TRS, middle panels **A** and **B**), where the zero point energy in the reactant and product wells are degenerate and the hydrogen can tunnel between the wells. Further heavy atom reorganization breaks the transient degeneracy and traps the hydrogen in the product state (bottom panels). The rate of reaching the TRS depends on the reorganization energy (λ) and driving force (Δ*G*°), which are indicated in the top panel (see [24] for more details and equations); (**C**) shows the effect of Donor–Acceptor Distance (DAD) sampling on the wave function overlap at the TRS (middle panel). Transmission probability (*P*) is a function of the overlap integral of the hydrogen wave functions in the reactant (blue) and product (red) wells (bottom panel **C**). The top panel **C** presents the contribution to H-transfer at each DAD as a function of the *P* and the population at each DAD. The vertical dashed line represents the DAD under which the Zero Point Energy (ZPE) is greater than the barrier height. At such distances, the process of a wave function spreading from reactant well to product well is no longer “tunneling”, but one can still use the particle’s transmission probability analogously to the tunneling probability at longer DADs.

In recent years, the KIEs and their temperature dependence have been used as a gold standard to probe the physical nature of enzyme- catalyzed H-transfer reactions in various studies [18,19,20,21,22]. Models that are used to rationalize temperature dependence of KIEs are sometimes called Marcus-like models [23,24,25,26]. In such a model (Figure 3), hydrogen transfers solely by nuclear quantum mechanical (QM) tunneling that is much faster than the motions of other atoms in the system (surrounding solvents and the protein environment). In the non-adiabatic limit, the reaction coordinate is comprised of two designate orthogonal coordinates, the heavy atom coordinate (Panel A) and the H-atom position (Panel B). Heavy atom reorganization brings the system to the tunneling ready state (TRS), which is the QM delocalized transition state and is the crossing point in middle panels A and B. When the system is at the TRS, hydrogen can tunnel between the donor and the acceptor.

In the non-adiabatic approximation (Figure 3), the heavy atom motion toward the TRS can be calculated as two parabolas (Marcus-parabolas). In such a case, the rate of reaching TRS is governed by the driving force (Δ*G*°) and the reorganization energy (λ) which are indicated in the top panel A. Please note that this term has little isotopic sensitivity to the mass of the particle being transferred, and thus it does not affect the model whether the system is electronically adiabatic or not. Panel C shows the effect of donor-acceptor distance (DAD) fluctuations on the wave function overlap at the TRS. This part is isotopically and temperature sensitive, but the difference between nuclear non-adiabatic H-transfer (tunneling) or the adiabatic one (over the barrier), is not substantial, and simply depends on the technical procedure used to calculate the mixing term between the reactant and product states. The top panel C presents a fraction of H-transfer events at each DAD as an integral of the transmission probability (*P*, bottom panel) and the population as function of DAD (middle panel). This model suggests temperature independent KIEs (as found for most natural, well-evolved enzymes) that result from TRS that is well defined. This means a narrow distribution of DADs, where the DAD’s sampling function (middle panel C) is of such high frequency that the integral is not affected by temperature (within the 35 °C range tested). For mutants [27], non-physiological substrates or conditions [28], or for primitive enzymes [29], a larger temperature dependence of the KIE_intS_ is interpreted as resulting from a broader DAD-distribution. Therefore, the temperature dependence of KIE_intS_ provides unique information on the chemical step of reactions.

In a recent study [15], we used both KIEs and X-ray crystallography experiments to study the correlation between the protein motions at various time scales that influence the hydride transfer reaction of the same TSase mutant Y209W under study here. It was found that with Y209W mutant, the reaction’s intermediate that precedes the hydride transfer forms a thiol-trapped by-product. This finding indicated that the mutation no longer forced the H-donor and acceptor close together, allowing small thiol to compete for that intermediate. Furthermore, the altered dynamics of the H-donor and acceptor at the TRS were probed by temperature dependence of KIE_int_, indicating disrupted DAD distribution.

Toward the broad goal of understanding in greater detail the impact of protein motions on different catalytic steps, we sought to study another C–H activation in the catalytic cycle of TSase, *i.e.*, the catalyzed proton transfer (step 4 in Scheme 1). In particular, we explore the kinetic properties of Y209W mutant further by comparing the temperature dependence of KIE_int_s on WT *vs.* Y209W-catalyzed proton transfer reactions. Along with the X-ray crystallographic data, we could correlate the kinetic findings and distinguish the effect of distal mutations on protein motions at different timescales that impact two different H-transfer reactions as well as other catalytic steps.

## 2. Results and Discussion

### 2.1. Binding Mechanism of Y209W Mutant

**Figure 4:**
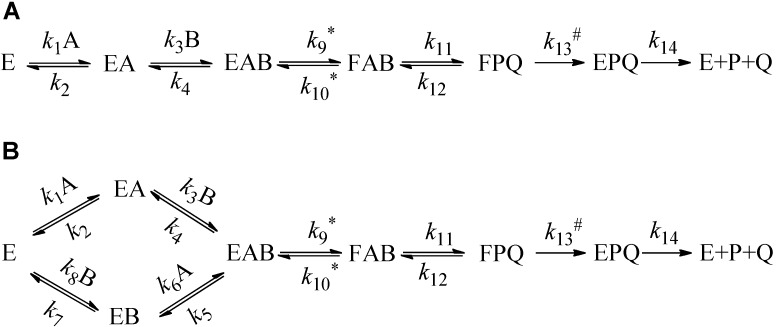
Binding Scheme for a Sequential Ordered Mechanism (**A**); and random binding mechanism (**B**). In the case of TSase, substrate A would be dUMP and B would be CH_2_H_4_folate. The rate constant with an asterisk represents isotopically sensitive steps in the proton abstraction experiments, using labeled dUMP and rate constants with hash signs is isotopically sensitive in the hydride transfer experiments using labeled CH_2_H_4_folate.

The wealth of information available for the WT *ec*TSase in terms of steady state kinetics, product inhibition studies, and structure suggests a bi–bi ordered mechanism of reactants binding and product release (Figure 4A) [13,30].

The observed KIEs on the second order rate constant (*V/K*_A_ or *V/K*_B_) for the individual substrates (A: dUMP and B: CH_2_H_4_folate in TSase) depend on the mechanism of the substrates’ binding with differences indicated in the expression for the forward commitment factor (*C*_f_, Equations (2)–(4)). The forward commitment factor is the ratio between the rate constants for the forward isotopically sensitive step and the isotopically insensitive steps proceeding backward. Therefore, the KIEs on *V*/*K* could discriminate the ordered and random sequential binding mechanisms [31,32]. However, the value of *V*/*K*_A_ is always dependent on the concentration of B, while the value *V*/*K*_B_ only depends on the concentration of A in the random mechanism (Figure 4B) but not in the ordered mechanism (Figure 4A). Therefore, we could use the substrate dUMP (A), labeled with tritium at C5 with varied concentrations of CH_2_H_4_folate, to distinguish between these two mechanisms.

To elucidate the binding mechanism of Y209W, we employed the following equation, which describes the relationship between the observed KIE (KIE_obs_) for H/T KIE_obs_ on *k*_cat_/*K*_M_ (^T^*V*/*K*) and the KIE after the formation of the ternary EAB complex in Figure 4 (H/T KIE on *k*_9_, or ^T^*k*_9_) [31,33].

(1) KIEobs=K9T+Cf+CrEIE1+Cf+Cr

where EIE (^T^*k*_eq._) is the equilibrium isotope effect on the proton abstraction step, *C*_f_ and *C*_r_ are the forward and reverse commitments to catalysis, respectively.

The *C*_r_ for the proton abstraction step represents the competition between the trace tritium and the water protons. Since the tritium released is diluted into ~100 M proton in the reaction medium, *C*_r_ is assumed to be zero. This is because the re-association of tritium is negligible given that its concentration is much less than that of protons in water. Thus, Equation (1) can be reduced to Equation (2):

(2) KIEobs=K9T+Cf1+Cf

In an ordered mechanism, Figure 4A, the commitment *C*_f_ is described by Equation (3):

(3) $$C_{\text{f}}=\frac{k_{9}(k_{2}+k_{3}\left[\text{B}\right])}{k_{2}k_{4}}$$

The commitment *C*_f_ for the Figure 4B is described by Equation (4):

(4) $$C_{\text{f}}=\frac{k_{9}}{k_{5}+\frac{k_{2}k_{4}}{k_{2}+k_{3}[\text{B}]}}$$

where [B] is the concentration of substrate B (CH_2_H_4_folate). For ordered mechanisms (Figure 4A), it is apparent from Equations (3) and (4) that *C*_f_ varies from infinity at infinite [B] to *k*_9_/*k*_4_ when [B] goes to zero. In the random mechanism (Figure 4B), *C*_f_ goes to *k*_9_/*k*_5_ at infinite [B] and to *k*_9_/(*k*_4_ + *k*_5_) at zero [B]. Consequently, the KIE_obs_ in the ordered mechanism vary between ^T^*k*_9_ at zero [B] to unity at high [B], while in the random mechanisms, KIE_obs_ vary between two finite values.

**Figure 5:**
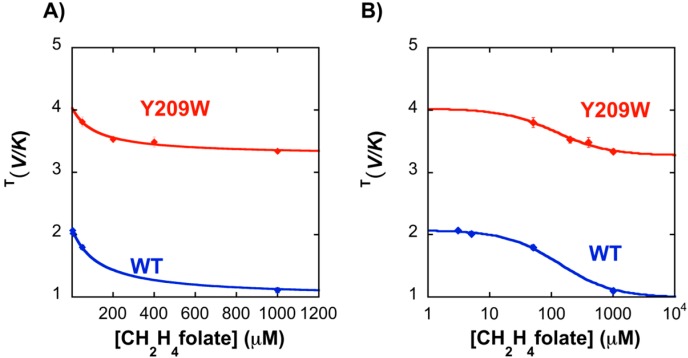
Observed H/T KIE for proton abstraction as a function of CH_2_H_4_folate concentration ((**A**) on linear scale and (**B**) on logarithmic scale). Data with WT (Blue) and Y209W (Red) are compared. The lines are fitted to Equation (2).

In the current study, KIE_obs_ on the proton abstraction step of the Y209W mutant was studied as a function of the concentration of CH_2_H_4_folate. As apparent from Figure 5, the KIE_obs_ for the WT approaches unity at higher concentrations (1 mM) of CH_2_H_4_folate, suggesting a strictly ordered binding mechanism in agreement with previous product inhibition and steady-state studies [34]. However, the binding mechanism for the Y209W mutant is more random, as suggested by the non-unity KIE at high CH_2_H_4_folate concentrations.

The observation that Y209W has a less ordered binding mechanism compared to WT *ec*TSase is further supported by the *K*_M_ values measured for the substrate and the cofactor for Y209W [15]. In Y209W, *K*_M_ for dUMP is increased by ~5-fold, and the *K*_M_ for CH_2_H_4_folate is increased by ~16-fold compared to WT, which is in accordance with the observed less ordered binding mechanism for the mutant.

### 2.2. Temperature Dependence of Intrinsic KIEs in the Y209W Mutant

**Figure 6:**
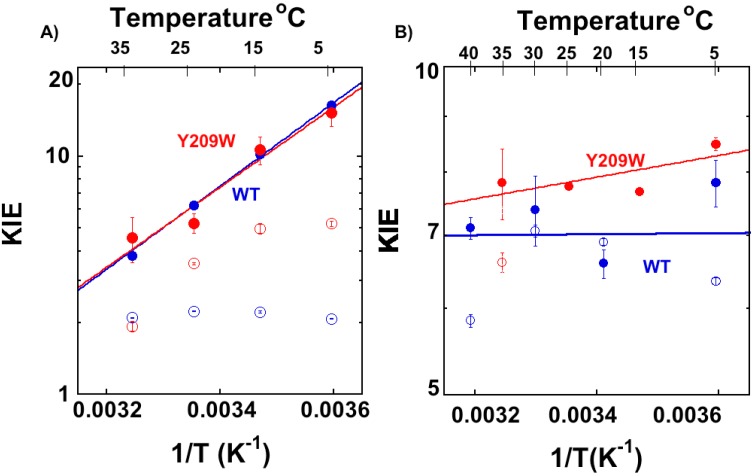
Arrhenius plots of the primary H/T KIEs on the proton abstraction (**A**) and the hydride transfer (**B**) catalyzed by the WT [16,25] (blue) and Y209W [15] (red). The empty circles represent the observed KIEs, and filled circles represent the intrinsic KIEs. The lines represent the least-squares nonlinear regression of KIE_intS_ to Equation (5). Figure S1 presents Arrhenius plots of both H/T and D/T KIEs on proton abstraction and hydride transfer steps.

To investigate the effects of Y209W mutation on the TRS of hydride and the proton transfer steps, we determined the temperature dependence of their intrinsic KIEs. We measured the observed KIEs on the second order rate constant (*V*/*K*) competitively and used the Northrop method to extract the KIE_intS_ as described before [32,35]. The KIE_intS_ are presented in Figure 6, where the lines represent the non-linear fit to the Arrhenius equation for KIE:

(5) $$\text{KIE}=\frac{k_{\text{L}}}{k_{\text{T}}}=\frac{A_{\text{L}}}{A_{\text{T}}}\text{exp}(\frac{-\Delta E_{\text{a}}}{\text{RT}})$$

where *k* is the microscopic rate constant of the isotopic sensitive step for the light (L) and heavy (T) isotopes, respectively; ∆*E*_a_ is the difference in the energy of activation between the light and heavy isotopes (∆*E*_a_ = *E*_aL_ − *E*_aT_); R is the gas constant; T is the absolute temperature; and *A*_L_/*A*_T_ is the isotope effect on the pre-exponential factors.

Table 1 summarizes the H/T and D/T isotope effects on the Arrhenius parameters of both the WT- and Y209W-catalyzed proton abstraction and hydride transfer steps. Importantly, the ∆*E*_a_ of WT and Y209W on hydride transfer are different, while upon proton transfer, they are similar within the experimental errors. Figure 6A shows the H/T KIEs on proton transfer and 6B the hydride transfer for both the WT (blue) and Y209W (red). A similar trend was observed for D/T KIEs and are presented in Figure S1 and the H/T and D/T KIE values on proton transfer is presented in Table S2.

**Table 1:** Isotope effects on Arrhenius parameters of wild type (WT) and Y209W *Escherichia coli* thymidylate synthase(*ec*TSase) on the proton abstraction and the hydride transfer.

	Proton Abstraction		Hydride Transfer	
	WT ^a^	Y209W	WT ^b^	Y209W ^c^
*A*_H_/*A*_T_	8.3 (±1.0) ×10^−6^	1.4 (±0.1) ×10^−6^	6.8 (±2.8)	3.6 (±0.9)
∆*E*_a H–T_ (kcal/mol)	−8.0 (±0.1)	−7.70 (±0.40)	−0.02 (±0.25)	−0.5 (±0.1)

^a^ Data from [16]; ^b^ Data from [25]; ^c^ Data from [15].

Apparently, the intrinsic KIEs on the hydride transfer of WT are temperature-independent while that of Y209W is more temperature-dependent [15]. The lack of temperature dependence of intrinsic KIEs is rationalized as a rearrangement of the heavy atoms (protein and solvent) toward narrow DAD distribution at the TRS. However, in the Y209W mutant, less perfect reorganization seems to occur, as reflected by KIEs with larger temperature dependence. The KIE_intS_ on the proton transfer, on the other hand, are similar and temperature dependent in both the WT and mutant. The fact that proton transfer has temperature dependent KIEs is in accordance with the faster proton transfer which lacks the enzyme induced “fine-tuning” that is critical for the hydride transfer. This has been rationalized as the differences between the difficulty to catalyze the hydride transfer and the ease of catalyzing the proton abstraction [16].

### 2.3. Effect of Y209W Mutation on Catalytic Steps Other than the Two C–H Activations

**Figure 7:**
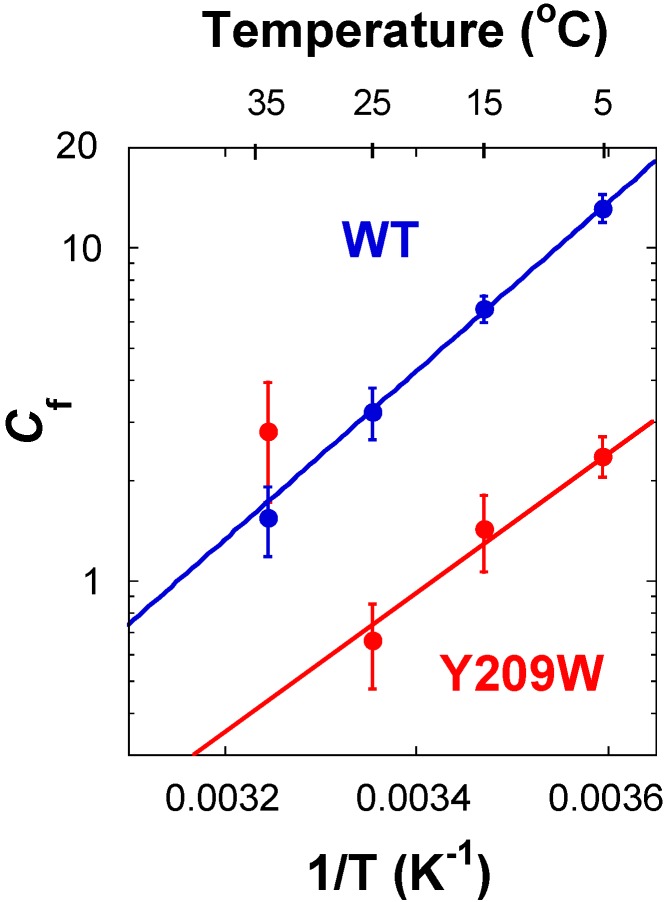
Comparison of commitment to catalysis (*C*_f_) on the *V*/*K* of proton abstraction for WT [16] (blue) and Y209W (red) *ec*TSase. The lines represent the exponential fit to all data points excluding Y209W at 35 °C.

For the proton transfer studied here and the hydride transfer at high and low temperatures, the observed KIEs are smaller than their corresponding intrinsic KIEs (Figure 7) due to kinetic complexity (*i.e.*, *C*_f_ > 0). However, the magnitudes of the observed KIEs on proton transfer are higher compared to WT, at the temperature range of 5–25 °C, indicating a reduced kinetic complexity in Y209W compared to WT, as evident from their prospective *C*_fS_.

Figure 6B reveals that there is no commitment on hydride transfer at 25 and 15 °C, indicating it is rate limiting for *V*/*K*. It is also true for *k*_cat_ at 25 °C as reported in [34]. The proton transfer, on the other hand, has non-zero commitments throughout the temperature range. Figure 7 presents the *C*_f_ values on the proton transfer of the WT and Y209W mutant as Arrhenius plots (logarithmic scale of *C*_f_ *vs.* the reciprocal of the absolute temperature). The exponential plots for the proton transfers suggest that essentially a single kinetic step is responsible for the *C*_f_, with the exception of 35 °C for the mutant.

At low concentrations of CH_2_H_4_folate, *C*_f_ for the proton transfer’s ^T^(*V*/*K*) approaches *k*_9_/(*k*_4_ + *k*_5_), *i.e.*, the ratio between the rates of the isotopically sensitive proton abstraction step and the rates of dissociation of either dUMP (A) or CH_2_H_4_folate (B) from the ternary enzyme–dUMP–CH_2_H_4_folate complex (the EAB complex in Figure 4). In addition, *C*_f_ on the hydride transfer’s ^T^(*V*/*K*) in the mutant case indicates that a step preceding the hydride transfer (most likely the proton abstraction) became more rate limiting. In contrast to the *C*_f_ of proton transfer with the WT, *C*_f_ with the mutant was significantly lower, which is in accordance with that step becoming more rate limiting overall.

## 3. Experimental Section

### 3.1. Materials

[2-^14^C] dUMP (specific radioactivity 53 Ci/mol) and [5-^3^H] dUMP (specific radioactivity 13.6 Ci/mmol) were from Moravek Biochemicals. Unlabeled CH_2_H_4_folate was a generous gift from Merck and Cie (Weisshausmatte, Switzerland). The WT and Y209W *ec*TSase enzymes were expressed and purified following a published procedure [36]. Ultima Gold liquid scintillation cocktails were from Perkin Elmer (Waltham, MA, USA). Liquid scintillation vials were from Research Products International Corp. (Mount Prospect, IL, USA). All other materials were purchased from Sigma (St. Louis, MO, USA). All the purifications and analytical separations were performed using an Agilent Technologies model 1100 HPLC system (Santa Clara, CA, USA). The column was from Supelco (C18, 250 × 4.6 mm, 5 μm, Discovery^®^; by Sigma, St. Louis, MO, USA). The radioactive samples were analyzed using a flow scintillation analyzer (Model RT505 from Packard, now Perkin Elmer Biosciences, Waltham, MA, USA) or a Liquid Scintillation Counter (LSC, from Perkin Elmer Biosciences, Waltham, MA, USA).

### 3.2. Synthesis of [2-^14^C,5-^2^H] dUMP(>99.5% D)

The synthesis of [2-^14^C, 5-^2^H] dUMP was carried out by the published procedure of Wataya and Hayatsu [37,38,39]. Briefly, the reaction mixture contained 1 M l-cysteine (pD = 8.8) and 1 mM [2-^14^C] dUMP in D_2_O solution (>99.96% D). The reaction mixture was incubated at 37 °C for 7 days until complete deuteration (>99.5% D) was achieved and verified by ^1^H NMR measurements.

### 3.3. Competitive KIEs on the Proton Abstraction (Step 4 in Scheme 1)

The competitive method for the primary H/T KIE on the proton transfer step was used according to the procedure published for the WT enzyme with modified experimental conditions [16,40]. The reaction mixture contained 100 mM tris(hydroxymethyl)aminomethane (Tris)/HCl buffer (pH = 7.5), 2 mM TCEP, 1 mM EDTA, 5 mM HCHO, 50 mM MgCl_2_, 0.5 Mdpm [2-^14^C] dUMP, and 1.5 Mdpm [5-^3^H] dUMP. Varied concentrations of CH_2_H_4_folate (50–1000 μM) were added to the reaction mixture and preincubated at 25 °C. All the experiments were performed following the previously published procedure with a modified HPLC method as given in Table S1. Three infinity time points (*t_∞_*) were obtained by adding concentrated WT TSase. Two independent time points with no enzymes (*t*_0_) were obtained as controls for the experiment. The competitive observed KIEs on the second order rate constant were calculated from the following equation [41].

(6) $$\text{KIE}=\frac{\text{ln}(1-f)}{\text{ln}(1-f\frac{R_{\text{t}}}{R_{\infty }})}$$

where *R*_t_ and *R*_∞_ are the ^3^H/^14^C ratio of products (^3^H_2_O and dTMP) at each time point and at the infinity time points respectively, and *f* is the fraction conversion typically ranging from 20% to 80%. The fraction conversion *f* was calculated by [25,40]:

(7) f=[C14]dTMP[C14]dTMP+ [C14]dUMP

To determine the intrinsic KIEs for the proton abstraction step, 200 μM CH_2_H_4_folate was used for both H/T and D/T KIE experiments at the desired temperatures at 5, 15, 25 and 35 °C. The observed D/T KIE was measured the exact same way except using [2-^14^C, 5-^2^H] dUMP instead of [2-^14^C] dUMP. The intrinsic KIEs for the proton abstraction step were calculated using the Northrop method as in Equation (8) [31,35,42]:

(8) (VK)Hobs−1T−1(VK)Dobs−1T−1=(kTkH)−1(kTkH)1/3.34−1

where *k*_i_ is the rate constant for the reaction involving isotope I and ^T^(*V*/*K*)_H__obs_ and ^T^(*V*/*K*)_D__obs_ are the observed competitive KIE values on the second order rate constant. Although *k*_T_/*k*_H_, the reciprocal of *k*_H_/*k*_T_ (intrinsic KIE) is the only unknown in Equation (8), it cannot be solved analytically. This equation was solved numerically using the program developed in our group. This program is available on our web site, http://cricket.chem.uiowa.edu/~kohen under “Tools”.

## 4. Conclusions

The role of long-range amino acid communications and enzyme dynamics across proteins and its catalytic function is of significant contemporary interest and controversy. In the current study, we examined a mutation Y209W *ec*TSase that is 8 Å from the site where chemistry took place in the enzyme active site. This mutant has been defined as a dynamically altered mutant, based on overlapping crystal structures (down to 1.3 Å resolution) with the WT enzyme and altered anisotropic B-factors. We compared the temperature dependence of intrinsic KIEs on two consecutive steps in the enzyme’s catalytic cycle: the fast and non-rate limiting proton abstraction (step 4 in Scheme 1), and the slow and mostly rate limiting hydride transfer step (step 5 in Scheme 1) of both the WT and Y209W mutant. The findings suggest that this dynamically altered mutant partly distorts the well-defined reactive state of the WT toward the hydride transfer but has no measurable effect on the proton abstraction. This finding can be rationalized in light of the fact that the hydride transfer is the most challenging chemical conversion in this system and has no relevant uncatalyzed equivalent model. Thus, the enzyme had to evolve for accurate rearrangement of the donor and acceptor toward the TRS and those dynamics involved Y209, as evident from the distorted TRS in the mutant.

The proton abstraction step, on the other hand, is not nearly as challenging following the Michael addition of Cys to C6 of the substrate (step 2 in Scheme 1). Indeed, we can easily mimic that step by incubating dUMP with a high concentration of cysteine in solution [37,38,39]. As the enzyme does not need to carefully orient the donor and acceptor in this case, the mutant’s altered dynamics have little to no effect on the nature of the H-transfer of that step. The comparison of intrinsic and observed KIEs, and the analysis of their kinetic complexity (via *C*_f_ calculations) indicated that other steps apart from the two C–H bond activations in question have also been altered. The commitments (*C*_f_ on *V*/*K*) of both H-transfers show a break above 25 °C, indicating an effect of Y209W that could be common to both steps. This observation would be in accordance with a step that precedes the proton transfer that is affected by the mutation. Such a step could be the release of CH_2_H_4_folate from the ternary complex, which would also be in accordance with higher *K*_M_ for that substrate, and the thiols attack on the dUMP intermediately prior to hydride transfer, indicating poor folate orientation in the active site.

In summary, despite its remote location relative to the site of chemical conversion catalyzed by this enzyme, the mutation of Y209 extends in the overall architecture of the enzyme, altering its dynamics (as indicated by anisotropic B-factors analysis), and affects several kinetic steps along the complex catalytic cascade of this enzyme. These results support a dynamic coupling of a remote residue to different kinetic events across the enzyme. The results further emphasize the importance of amino acid residues controlling long-range protein dynamics critical for the enzyme function.
