# Supplementary material for: Role of Long-Range Protein Dynamics in Different Thymidylate Synthase Catalyzed Reactions
Source: Int J Mol Sci. 2015 Apr 1;16(4):7304–19. doi: 10.3390/ijms16047304 (PMC4425018; doi:10.3390/ijms16047304)
Supplement: Supplementary file 1 [file ijms-16-07304-s001.pdf]

Supplementary Information

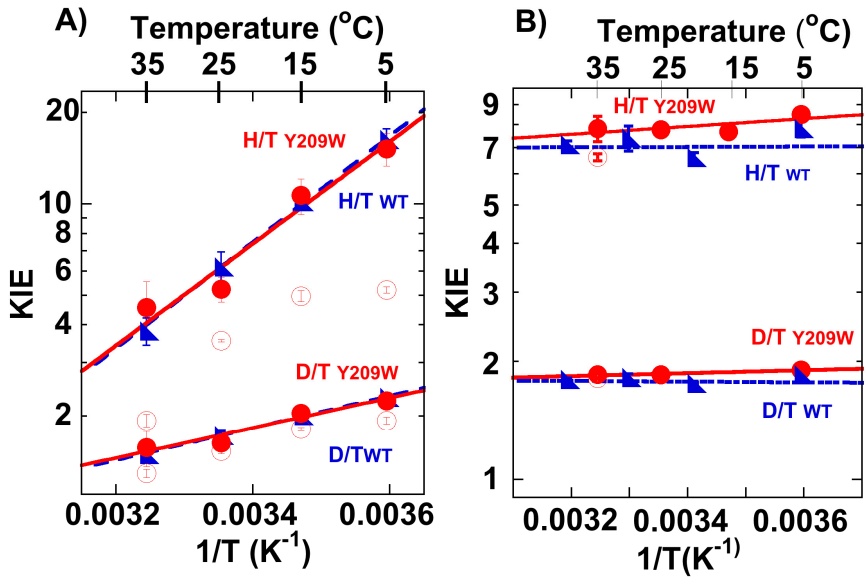

Figure S1. Arrhenius plots of the primary KIEs on the proton abstraction (**A**) catalyzed by WT [16] (blue) and Y209W (red) TSase and the hydride transfer (**B**) catalyzed by the WT [25] (blue) and Y209W [15] (red) TSase. The lines represent the least-squares nonlinear regression of the intrinsic KIEs to Equation (5) in the main text. The empty circles represent the observed KIEs, and filled circles represent the KIE_{int}s. (**B**) is adapted from [15] with permission from ACS.

Analysis of Products in the Proton Abstraction Step

We used an Agilent Technologies model 1100 HPLC system with a Supelco Discovery® C18 reverse phase column.

HPLC mobile phase solutions: A = 20 mM TEAA buffer (pH = 5.0); B = Acetonitrile.

Table S1. HPLC mobile phase gradient (flow rate 0.8 mL/min).

Time (min)	% A	% B
0	100	0
5	99.5	0.5
20	99	1
25	90	10
30	50	50
35	50	50

Table S2. KIE on the proton transfer catalyzed by Y209W.

**Temperature (°C)**	**Observed KIE**		**Intrinsic KIE**	
	**H/T KIE**	**D/T KIE**	**H/T KIE**	**D/T KIE**
5	5.21 ± 0.13	1.93 ± 0.05	15.19 ± 1.86	2.24 ± 0.13
15	4.97 ± 0.21	1.81 ± 0.02	10.67 ± 1.43	2.04 ± 0.1
25	3.54 ± 0.05	1.53 ± 0.03	5.24 ± 0.48	1.63 ± 0.04
35	1.93 ± 0.09	1.29 ± 0.04	4.56 ± 0.99	1.57 ± 0.21
